# Complete Genome Sequence of *Paenibacillus* sp. Strain IHBB 10380 Using PacBio Single-Molecule Real-Time Sequencing Technology

**DOI:** 10.1128/genomeA.00356-15

**Published:** 2015-04-23

**Authors:** Mohinder Pal, Mohit K. Swarnkar, Rishu Thakur, Shashi Kiran, Sanjay Chhibber, Anil K. Singh, Arvind Gulati

**Affiliations:** a CSIR-Institute of Himalayan Bioresource Technology, Palampur, India; b Department of Microbiology, Panjab University, Chandigarh, India

## Abstract

The complete genome sequence of 5.77 Mb is reported for *Paenibacillus* sp. strain IHBB 10380, isolated from the cold desert area of the northwestern Himalayas and exhibiting amylase and cellulase activities. The gene-coding clusters predicted the presence of genes for hydrolytic enzymes in the genome.

## GENOME ANNOUNCEMENT

The genus *Paenibacillus* has been separated from the genus *Bacillus* based on the phylogenetic analysis of the 16S rRNA gene to accommodate the aerobic or facultatively anaerobic, rod-shaped, and endospore-forming bacilli (1). More than 161 species of *Paenibacillus* have been reported from diverse habitats (http://www.bacterio.net/paenibacillus.html). Several species of the genus have been reported for the production of biotechnologically important hydrolytic enzymes, such as cellulases (2), xylanases (3), amylases (4), and glycanases (5). In our study, a novel species named *Paenibacillus* sp. strain IHBB 10380 has been isolated from the soil of the forefield of a glacier near the Kunzum Pass (32°23′45.93″ N 77°37′46.00″ E) in the Lahaul-Spiti district of Himachal Pradesh. The cells of the strain are aerobic, Gram-positive, motile, and endospore-forming rods, which showed hydrolysis zones on starch agar for amylase activity (6) and on carboxymethyl cellulose agar for cellulase activity (7).

The genomic DNA was isolated by using the phenol-chloroform-isoamyl alcohol extraction procedure from a 72-h-old culture of *Paenibacillus* sp. strain IHBB 10380 grown at 28°C in trypticase soya broth shake flasks at 200 rpm (8). The quantity and quality of extracted genomic DNA were determined by a Qubit version 2.0 fluorometer (Invitrogen, USA) and NanoDrop 2000 (Thermo Scientific, USA), respectively. Genomic DNA was sheared to 10 kb using Covaris g-tubes (Covaris, USA) to construct the DNA library. The quality of the sheared DNA was checked with a Bioanalyzer DNA 12000 chip (Agilent Technologies, USA). A PacBio SMRTbell library preparation kit version 1.0 was used to prepare the genomic DNA library per the manufacturer’s instructions. The prepared SMRTbell library was quantified, and sequencing was performed on a PacBio RSII system on two SMRT cells using P5 polymerase and C3 sequencing chemistry with a 180-min movie time. The two SMRT cells produced 1,447,995,075 nucleotide bases generated through 383,458 reads (*N*_50_ size 4,915 and mean subread length 3,776). The generated subreads were *de novo* assembled using the RS hierarchical genome assembly process (HGAP) protocol version 2.0 in SMRT Analysis version 2.2.0 (Pacific Biosciences, USA) and yielded a gapless, complete circular genome sequence with 202-fold coverage (9).

The functional annotation was performed with the Rapid Annotations using Subsystems Technology (RAST) server (10), which predicted 5,861 genes, 5,638 protein-coding genes (CDSs), 108 tRNAs, and 125 rRNAs. The genome size is 5,770,242 bp with a GC content of 41.33%; a 17-kb plasmid (39.18% GC content) was also identified.

A comparison of the *Paenibacillus* sp. IHBB 10380 genome sequence with the existing genome sequences was performed using the RAST server, which revealed *Geobacillus* sp. Y412MC10 (score 534) and *Paenibacillus* sp. oral taxon 786 strain D14 (score 492) as the closest neighbors. The annotation also predicted gene clusters coding for the hydrolytic enzymes of O-glycosyl hydrolase (one gene), glycoside hydrolase (five genes), and β-glucosidase (five genes) families, which have been reported for application in the food, pharmaceutical, textile, paper, and detergent industries (11, 12).

### Nucleotide sequence accession numbers. 

The complete genome sequence and the plasmid sequence from *Paenibaciillus* sp. IHBB 10380 have been deposited at DDBJ/EMBL/Genbank under the accession numbers CP010976 and CP010977, respectively.
